# Case report: Improved behavioral and psychiatric symptoms with repetitive transcranial magnetic stimulation at the bilateral DLPFC combined with cognitive and behavioral therapy in a patient with unilateral thalamic hemorrhage

**DOI:** 10.3389/fneur.2022.880161

**Published:** 2022-07-25

**Authors:** Hye Chan Ahn, Kyoung Tae Kim

**Affiliations:** Department of Rehabilitation Medicine, Keimyung University Dongsan Hospital, Keimyung University School of Medicine, Daegu, South Korea

**Keywords:** thalamic stroke, repetitive transcranial magnetic stimulation, dorsolateral prefrontal cortex, intervention, cognitive-behavioral therapy

## Abstract

Behavioral and psychological symptoms are not uncommon after thalamic stroke, and are often intractable despite medication and behavioral interventions. Repetitive transcranial magnetic stimulation (rTMS) is as an adjunctive therapeutic tool for neuropsychiatric diseases, and bilateral rTMS has been recently introduced to maximize the therapeutic effect. Herein, we report the case details of a patient with unilateral left thalamic hemorrhage without cortical lesions who had treatment-resistant neuropsychiatric symptoms. We hypothesized that bilateral rTMS targeting the bilateral dorsolateral prefrontal cortices (DLPFCs) would positively affect thalamocortical neural connections and result in neuropsychiatric symptom improvement. The patient received a total of 10 sessions of bilateral rTMS over 2 weeks, applied at the DLPFCs, with high frequency in the left hemisphere and low frequency in the right hemisphere. After each rTMS treatment, computer-based cognitive-behavioral therapy was administered for 30 min. Behavioral and psychological symptoms, including hallucinations, aggressiveness, aberrant motor activity, disinhibition, and abrupt emotional changes, were significantly improved as assessed by the Neuropsychiatric Inventory Questionnaire. These effects persisted for up to 1 month. This case demonstrates the clinical potential of bilateral rTMS treatment in patients with intractable neurocognitive impairment after thalamic stroke.

## Introduction

Thalamic stroke is not a rare disease, and can occur in isolation or in combination with other structural involvements (1, 2). It presents with various symptoms, depending on its location, volume, and lateralization, and can affect memory, emotions, the sleep-wake cycle, general cortical alerting responses, sensory processing, sensorimotor control, and the relay of information to the cortex (3). Regarding lesion-based neuropsychiatric symptoms after thalamic stroke, tuberothalamic lesions deteriorate arousal, orientation, learning, memory, personality, and executive function (4). Bilateral paramedian lesions cause decreased arousal, learning, and memory (4). Although neuropsychiatric symptoms and neurocognitive deterioration are prominent in patients with bilateral thalamic stroke, symptoms can also persist in unilateral lesions (5).

Pharmacological management and behavioral interventions are still mainstream treatments for neuropsychiatric symptoms in patients with thalamic stroke. However, many patients do not improve with such treatments. Non-invasive brain stimulation (NIBS) is an effective adjunctive therapy for treatment-resistant neuropsychiatric symptoms (6). Repetitive transcranial magnetic stimulation (rTMS), a type of NIBS that has been widely used in the neurorehabilitation field (7) has emerged as a therapeutic tool to facilitate neuroplasticity, with clinical benefits in neuropsychiatric diseases. Stimulation targeted to the dorsolateral prefrontal cortex (DLPFC) has been proven as clinically effective in mild cognitive impairment, obsessive-compulsive disorder, and major depressive disorder (6, 8). Furthermore, in patients with stroke, rTMS has shown promising results in improving cognitive impairments and mood disorders (9–12). Recently, bilateral rTMS was introduced to maximize the therapeutic effectiveness (13–15). However, there is a lack of consensus regarding the intensity and frequency of the rTMS protocol, and no previous studies have applied bilateral rTMS to treat stroke-related neuropsychiatric symptoms.

Here, we report a case of unilateral thalamic stroke with treatment-resistant neuropsychiatric symptoms, improved by the application of bilateral rTMS at the bilateral DLPFCs, with high frequency in the left hemisphere and low frequency in the right hemisphere.

## Case presentation

**Figure 1:**
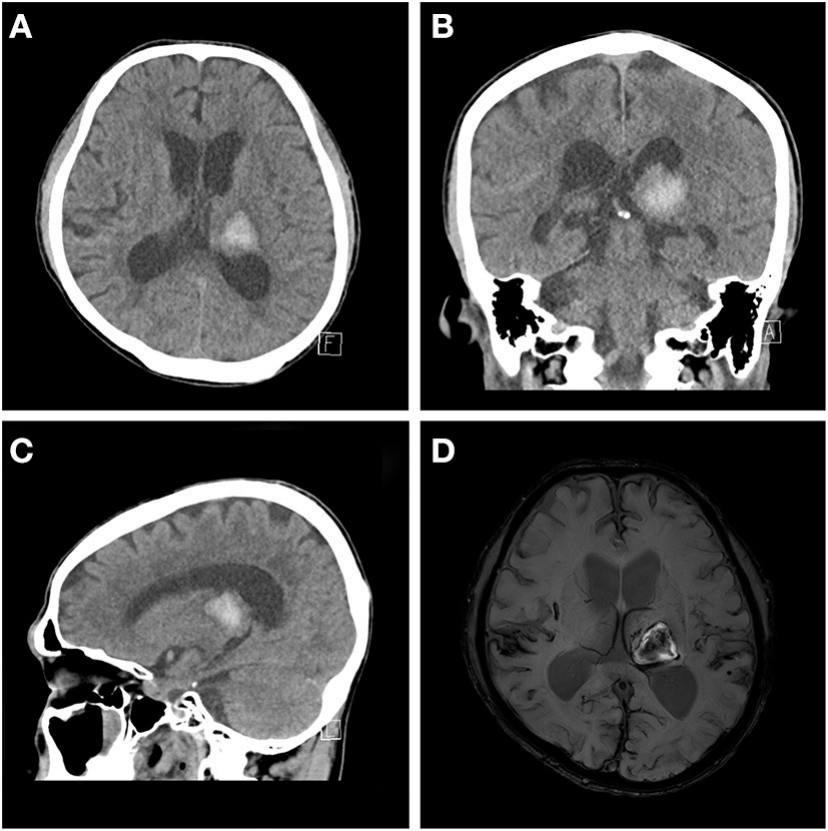
Baseline brain magnetic resonance imaging and computed tomography (CT) scan. Acute intracerebral hemorrhage at the left thalamus is observed on axial **(A)**, coronal **(B)**, and sagittal views **(C)** of the brain CT scan and the axial view of the diffusion-weighted image **(D)**.

A 63-year-old man was admitted to our hospital with left thalamic hemorrhage. He was in a comatose state when he arrived to the emergency room. His initial Glasgow Coma Scale (GCS) score was 7, but his consciousness level rapidly improved to a GCS score of 14. He had subjective right-sided weakness, but no obvious motor impairment was observed on physical examination. Rehabilitative intervention was conducted focused on locomotion and activities of daily living rather than limb weakness. Computed tomography (CT) revealed a left thalamic hemorrhage with intraventricular hemorrhage (IVH). He received conservative treatment with blood pressure control. Serial follow-up brain CT revealed a newly developed, tiny IVH, but no other hemorrhage. Brain magnetic resonance imaging (MRI) revealed moderate small-vessel disease in the white matter and amyloid angiopathy with diffuse mild cortical atrophy (Figure 1). Although his alertness rapidly improved, he showed fluctuations in consciousness with delirium at night, disorganized thought, inattention, and perceptual disturbances. Antipsychotics were administered, but these symptoms did not improve.

Three weeks after stroke onset, the patient was referred to our rehabilitation department for cognitive rehabilitation. Neurocognitive assessment revealed various behavioral and psychological symptoms. Long-term memory was relatively spared, but short-term memory was markedly reduced. Orientation in space, time, and person was severely impaired. Visual hallucinations of something crawling on the curtains and abrupt changes in emotional status were also observed. The Neuropsychiatric Inventory–Questionnaire (NPI-Q) indicated moderate to severe symptoms, and moderate caregiver distress. His scores on the Korean version of the Mini-Mental Status Exam (K-MMSE); score: (12), Clinical Dementia Rating (CDR); global score: 3; sum of box score: (13), and the Korean version of the Montreal Cognitive Assessment (MOCA-K); score: (2) indicated severe neurocognitive impairment (Table 1). We attempted to administer an overall cognitive assessment battery, but this was impossible because of the patient's inattention and perseveration.

**Table 1:** Change in neurocognitive assessment scores after repetitive transcranial magnetic stimulation.

	**Before treatment**		**Immediately after treatment**		**1 month after treatment**	
**K-NPIQ**	
	**Severity**	**Distress**	**Severity**	**Distress**	**Severity**	**Distress**
Delusions	NA		NA		NA	
Hallucinations	2	3	1	1	1	1
Agitation	3	3	2	2	NA	
Depression	2	2	1	1	2	1
Anxiety	2	2	1	1	1	1
Euphoria	1	2	1	1	1	1
Apathy	3	2	1	1	1	1
Disinhibition	3	2	1	1	1	1
Irritability	3	4	1	1	1	1
Aberrant motor activity	3	3	1	1	1	1
Nocturnal aberrant activity	NA		NA		NA	
Prandial aberrant activity	NA		NA		NA	
Total	22	23	10	10	9	8
MOCA-K	2		7		6	
CDR			
Global score	3		3		2	
Sum of box	15		15		12	
K-MMSE	12		14		14	

*NA, not applicable; K-NPIQ, Korean Neuropsychiatric Inventory-Questionnaire; MoCA-K, Korean version of Montreal Cognitive Assessment; CDR, Clinical Dementia Rating; K-MMSE, Korean version of Mini-Mental State Examination*.

A N-methyl-D-aspartate antagonist (NMDA) receptor antagonist (memantine, 10 mg) and anti-depressant (escitalopram 10 mg) were administered to improve his attention and emotional lability, and a small amount of atypical antipsychotics (quetiapine, 12.5 mg) was administered to alleviate his delirium. A neuro-stimulant (methylphenidate) was also administered to improve his attention, but this was stopped because his irritability was aggravated. He received computer-based cognitive-behavioral therapy (CCBT) using CoTras (Netblue Co., Ltd, Korea) software (16) for 3 weeks. However, there was no noticeable change in his cognitive function and neuropsychiatric symptoms. Thus, we decided to apply bilateral rTMS to the DLPFCs, with the consent of the patient and caregiver.

### rTMS protocol

**Figure 2:**
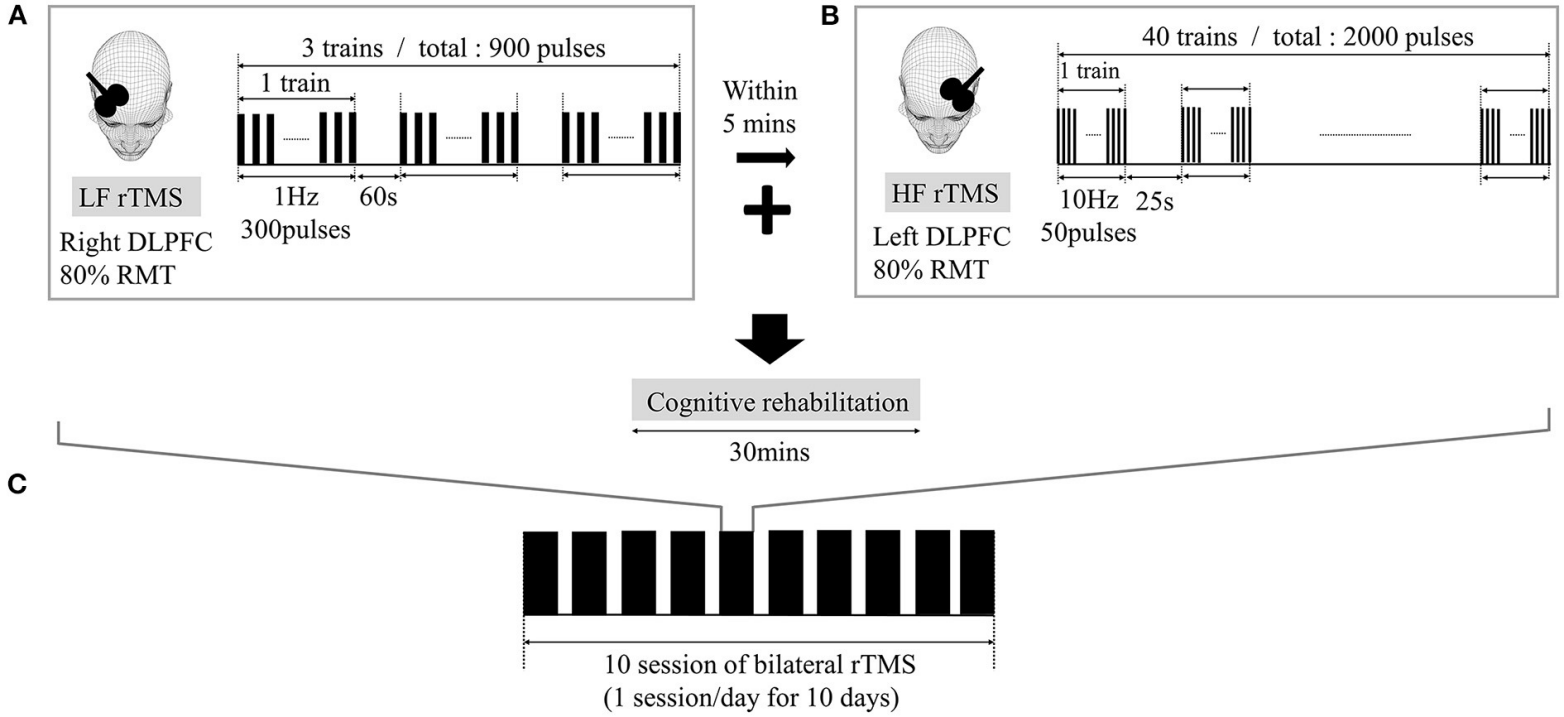
Repetitive transcranial magnetic stimulation (rTMS) montage and intervention protocol. High-frequency rTMS was applied to the left dorsolateral prefrontal cortex (DLPFC) and low-frequency rTMS was applied to the right DLPFC **(A,B)**. Low-frequency rTMS was applied first, followed by high-frequency rTMS during each session, and a total of ten sessions were performed **(C)**.

rTMS was performed with a Magstim Super Rapid Stimulator (Magstim Co., United Kingdom) with a 70-mm, figure-eight shape, air cooled coil. The handle was oriented posteriorly, with a 45° angle sagittally. Single-pulse transcranial magnetic stimulation was conducted at the bilateral primary motor cortices to identify the motor hot spot in each hemisphere. The motor-evoked potentials (MEPs) were recorded in the contralateral abductor pollicis brevis, and the motor threshold (MT) was defined as the stimulus intensity required to provoke MEPs of >50 μV in peak-to-peak amplitude, in five of 10 sequential trials. The stimulation target of the DLPFC was defined as 5 cm anterior to the motor hot spot, parallel to the sagittal midline (17). For low-frequency rTMS, 1-Hz stimulation at 80% MT was applied in three trains of 5-min duration each, with a 1-min inter-train interval, and a total of 900 pulses (a total period of 20 min). For high-frequency rTMS, 10-Hz stimulation at 80% MT was applied in 40 trains of 5-s duration each, with a 25-s inter-train interval, and a total of 2,000 pulses (a total period of 20 min). Low-frequency rTMS was applied to the right DLPFC, followed by high-frequency rTMS to the left DLPFC, with a 5-min pause. CCBT was administered within 30 min after rTMS treatment. A schematic of the treatment protocol is provided in Figure 2. During the rTMS treatment period, the existing medications (memantine, escitalopram, quetiapine) were continuously used.

Bilateral rTMS was applied 53 days after onset of thalamic hemorrhage followed by a total of 10 sessions over 2 weeks. There were no adverse events, such as headaches, seizures, and other neurologic deficits, and there was only mild scalp discomfort during the treatment sessions. Behavioral and psychological symptoms were markedly improved, as assessed by the K-NPIQ (Table 1). Both symptom severity and caregiver distress were improved in all domains, with the exception of delusions, a nocturnal and prandial aberrant activity that was not observed, even before treatment. The MOCA-K score also improved. However, amnestic symptoms were only slightly enhanced. These effects of rTMS persisted for 1 month after treatment.

## Discussion

To our knowledge, this is the first case report of bilateral rTMS to the DLPFCs in a patient with behavioral and psychological symptoms after thalamic stroke. This patient showed a clear clinical response to bilateral rTMS treatment targeting the bilateral DLPFCs. Hallucinations, agitation, irritability, and anxiety were significantly improved after the treatment, and these effects remained after 1 month. We found that these behavioral and psychological symptoms were the main causes of caregiver distress. In contrast, working memory and executive function were not improved. There were no adverse events associated with bilateral rTMS.

The thalamus is recognized as one of the major cognitive centers for neural processing, and routes information across the brain in cortico-cortical, cortico-striatal, hippocampo-cortical, and cerebello-cortical pathways (18). There are numerous neuroscientific studies on contributions from different parts of the thalamus to cognitive functions in non-human primates. In memory and learning processes, the anterior thalamus is particularly driven by the hippocampus, and interacts with the cortex in memory processing and spatial navigation in rodent studies (19). Minamimoto et al. showed that the intralaminar thalamus interacts with the basal ganglia, and contributes to counteracting behavioral biases, enabling behavioral flexibility (20). The mediodorsal thalamus is a component in a neural circuit involving the prefrontal cortex that has a crucial role in spatial working memory which enables the transformation of retrospective information into prospective information (21, 22). It also shows preferential connectivity with the DLPFC, as demonstrated in a previous tractography study (23). Strong relationships between the cortex and thalamus have also been shown in human research. Behrens et al. revealed a specific connection between the human thalamus and cortex using quantitative diffusion imaging data (24). Furthermore, in a positron emission tomography study, significant ipsilateral hypometabolism was observed in the cortex of patients with neuropsychological deficits after vascular thalamic injury (25).

Among the NIBS techniques for neurocognitive disorders, deep brain stimulation can directly stimulate deep neural structures, and has proven to be effective when targeted to subgenual regions in mood disorders and the thalamus in dystonia, Parkinson's disease, and essential tremor (26–29). rTMS is a NIBS technique based on producing a rapidly shifting magnetic field over the scalp, which induces an electric current in the cortex parallel to the magnetic coil. It can modulate the neuronal excitability of the cortical surface directly underneath the coil and associated other brain regions (30). Low frequency rTMS (1 Hz) reduces cortical excitability, whereas rTMS at high frequency (10 Hz) facilitates neuronal excitability. Unfortunately, rTMS is largely limited to the cortical surface and deeper neural structures, such as the thalamus, cannot be selectively and directly stimulated. Therefore, in neuropsychiatric situations, the DLPFC is the most commonly used therapeutic target for rTMS, based on its importance in neural networks.

In post-stroke rehabilitation, rTMS has been shown to be a safe and well-tolerated intervention, and has been recommended as a viable therapy to enhance clinical recovery and functional improvement (31). Evidence-based guidelines suggested the definite efficacy of LF-rTMS of contralesional M1 and probable efficacy of HF-rTMS of ipsilesional M1 in hand motor recovery (32). Moreover, they reported clinical effectiveness in post-stroke apahsia and hemispatial neglect. Several studies have shown evidence of rTMS targeting DLPFC in post-stroke depression and cognitive impairment (9, 33).

DLPFC is the most frequently used stimulation target for post-stroke non-motor symptoms, and evidence of a functional connection between the thalamus and DLPFC has been reported recently. Neurophysiologic study using short-latency afferent inhibition revealed the direct thalamocortical connectivity and highlighted its importance as a marker of cognitive and behavioral activity in the neurorehabilitation field (34, 35). Li et al. also showed the functional connectivity between DLPFC and thalamus. A single session of active rTMS at DLPFC inhibits brain activity of the thalamus in fractional amplitude of low frequency fluctuation (36). It has been hypothesized that rTMS targeting the DLPFC might affect deeper regions that share the same neural pathway (7, 8). We also hypothesized that DLPFC rTMS positively affects thalamocortical neural connections, resulting in neuropsychiatric symptom improvement in unilateral isolated thalamic stroke without cortical lesions.

Stimulation localization is the crucial component to enhance the efficacy of rTMS. The “5 cm rule” introduced by Pascual-Leone et al. was widely used in early research trials to localize the DLPFC (37). The motor hotspot for the contralateral abductor pollicis brevis muscle is first identified during motor evoked potential testing, and then a target site is defined 5 cm anteriorly to this site for DLPFC stimulation. Neuro-navigation system based on structural brain MRI to find DLPFC as the boundary between BA 9 and 46 seems to be the most reasonable method (38). But it is challenging because of technical difficulty and cost. Other alternative methods such as F3/4 EEG location based on standard 10–20 system, Beam F3 method, and neuro-cardiac-guided TMS have been introduced recently (39). Since we localized DLPFC using the conventional 5-cm rule without using a navigation system, there were limitations in obtaining better therapeutic effects.

In neuromodulation using rTMS, besides the stimulation location, it is also very important to establish a protocol including frequency, intensity, time interval of stimulation, and total pulses with sessions. It is challenging to establish physiologic evidence for setting the value with the best therapeutic effect in each parameter. Therefore, we designed our protocol based on well-designed previous randomized controlled studies, in which rTMS was performed targeted to DLPFC in patients with post-stroke cognitive and mood disorders (11, 12).

Bilateral rTMS has been scrutinized as a novel approach in recent studies. Khedr et al. reported that a 10-day protocol of low-frequency (1 Hz) rTMS over the unaffected right Broca's area with 1,000 pulses, followed by 20-Hz high-frequency rTMS over the affected left Broca's area with 1,000 pulses resulted in language function improvement in non-fluent aphasia (13). In a study by Fitzgerald et al., 6 weeks of 1-Hz rTMS to the right DLPFC with 430 pulses, followed by 10-Hz rTMS to the left DLPFC with 750 pulses was compared with sham stimulation for 6 weeks; a marked benefit of bilateral rTMS in intractable depression was demonstrated (15). Based on these previous studies, we applied high frequency rTMS within 5 min after low frequency rTMS of 1Hz.

The patient in the present case report did not show any improvement in neuropsychiatric symptoms after 2 weeks of behavioral interventions and pharmacological treatment. Neurostimulants could not be continued because of adverse events. Therefore, to maximize the treatment effect, we applied sequential bilateral rTMS, with combined high-frequency and low-frequency rTMS. A few studies have examined the effects of rTMS on neurocognitive capacity after stroke and highlighted its positive effects on cognitive function and daily activities (9, 10, 12). After 2 weeks of rTMS treatment, hallucinations, aggressiveness, aberrant motor activity, disinhibition, and abrupt emotional changes were markedly improved, and this improvement lasted for a month. Consistent with our findings, rTMS has shown a positive effect on hallucinations in schizophrenia (40), disinhibition in obsessive-compulsive disorder (41), and nicotine dependence (42). Unfortunately, the decline in cognitive functions, including working memory, orientation, and executive function, did not improve.

There are some limitations to generalizing the effect of bilateral rTMS based on this study. Since it was an acute stage after stroke, improvement of symptoms due to spontaneous recovery could not be ruled out. In addition, although medication was unavoidably used to control the patient's symptoms, the continued use of medicines that affect brain activity during the rTMS treatment session may have affected the results.

## Conclusion

This is the first report of successful bilateral rTMS treatment in a patient with neurocognitive impairment due to thalamic stroke. Thalamic stroke causes behavioral and psychological symptoms, which are often intractable despite medication or behavioral interventions. This case study supports research opportunities for the therapeutic use of rTMS for treatment-resistant neuropsychiatric symptoms after thalamic stroke. Future studies are needed to evaluate the impact of rTMS on neurocognitive impairment in large cohort groups. More evidence for the effect of rTMS on thalamocortical connections should be established.

## Data availability statement

The original contributions presented in the study are included in the article/supplementary material, further inquiries can be directed to the corresponding author.

## Ethics statement

The studies involving human participants were reviewed and approved by the Institutional Review Board (IRB) of Keimyung University Dongsan Hospital (IRB No: 2021-11-062). The patients/participants provided their written informed consent to participate in this study. Written informed consent was obtained from the participant for the publication of this case report.

## Author contributions

HCA designed and implemented the data analysis and wrote the manuscript. KTK participated in data interpretation, analysis, and design of the rTMS protocol. Both authors participated in editing. All authors contributed to the article and approved the submitted version.

## Conflict of interest

The authors declare that the research was conducted in the absence of any commercial or financial relationships that could be construed as a potential conflict of interest.

## Publisher's note

All claims expressed in this article are solely those of the authors and do not necessarily represent those of their affiliated organizations, or those of the publisher, the editors and the reviewers. Any product that may be evaluated in this article, or claim that may be made by its manufacturer, is not guaranteed or endorsed by the publisher.
